# What if I needed help? Population preferences for first-line mental health treatments in the post-COVID time

**DOI:** 10.3389/fpsyt.2026.1843041

**Published:** 2026-05-28

**Authors:** Ulrich Voderholzer, Eva P. Wuttke, Eva M. Zisler, Anna Baumeister, Lena Jelinek

**Affiliations:** 1 Department of Psychiatry and Psychotherapy, LMU University Hospital, LMU Munich, Munich, Germany; 2 Schoen Clinic Roseneck, Prien am Chiemsee, Germany; 3 Department of Psychiatry and Psychotherapy, University Hospital of Freiburg, Freiburg, Germany; 4 Department of Psychology, University of Regensburg, Regensburg, Germany; 5 Department of Psychiatry and Psychotherapy, University Medical Centre Hamburg-Eppendorf, Hamburg, Germany

**Keywords:** common mental disorders, population survey, psychopharmacology, psychotherapy, treatment preferences

## Abstract

**Aims:**

Treatment guidelines for mental disorders recommend psychotherapy, psychopharmacology, or both as first-line treatments. However, it remains unclear to what extent these recommendations align with public treatment preferences. While treatment preferences are central in patient-centred care, it remains unclear, whether persons would choose recommended first-line treatments. Understanding these preferences may reveal gaps between recommendations and public perception.

**Methods:**

2,125 participants (48% female) in Germany completed an online survey about preferred treatment modalities in the case of a personal mental disorder. Linear-mixed effects models examined variation in preferences across disorders. *Post-hoc* comparisons contrasted psychotherapy and psychopharmacology across disorders. Sociodemographic and clinical predictors were examined.

**Results:**

About 36% of respondents reported previous treatment experience. The majority preferred psychotherapy over psychopharmacology. While psychotherapy was favoured across different mental health conditions, the extent of this preference varied depending on the specific disorder. The strongest preference for psychotherapy over psychopharmacological treatment was observed for depression, whereas the smallest difference was found for psychotic disorders, despite the central role of pharmacological treatment. Participants who were female, had prior treatment experience, or were employed in the healthcare sector were less likely to prefer pharmacological treatment.

**Conclusions:**

The findings reveal a strong preference for psychotherapy over psychopharmacological treatment and highlight discrepancies between guideline-recommended treatments and public preferences, the underlying reasons of which were not examined in the present study. Increasing the availability of psychotherapy, in line with both guidelines and public preferences, appears warranted.

## Introduction

Evidence-based guidelines have become essential tools to translate scientific knowledge into consistent and effective mental health care. These guidelines synthesize the best available research evidence and offer structured recommendations regarding first-line treatment options, such as psychotherapy, psychopharmacology, or a combination of both. However, a key limitation is that guideline recommendations are often based on short-term randomized controlled trials in selected populations, while long-term outcomes and side effects of continued treatment are often insufficiently studied, and in clinical practice treatment is not always delivered in accordance with these guidelines (1–3).

A meta-analysis by Setkowski, Boogert (4) demonstrated that guideline-based treatment outperform treatment as usual, with moderate effect sizes in favour of guideline-conform approaches – though most included studies focused on short-term outcomes. Importantly, clinicians adhering closer to guideline recommendations achieved significantly better patient outcomes, although additional factors such as patient factors or factors related to the organisation of care may also have contributed to these improvements (1). Furthermore, guideline-based treatment led to significantly faster remission than treatment as usual. As such treatment guidelines represent a structured and evidence-informed foundation for clinical decision-making. For most mental disorders the guidelines recommend psychotherapy (5–13) while for psychotic disorders (especially schizophrenia), a combination of psychotherapy and psychopharmacology is typically advised (14, 15). However, it remains unclear whether these guideline-based recommendations are aligned with public treatment preferences for specific mental disorders.

The effective implementation of such guidelines depends not only on provider adherence but also on the engagement and acceptance of patients. Increasingly, the importance of incorporating patient preferences into treatment planning has been recognized as a key component of patient-centred care and is also a core element of evidence-based practice (16). In a recent qualitative study most patients reported that accommodating preferences enhanced the therapeutic relationship and intrapersonal outcomes, however some (5 of 13) also experienced benefits from working through unmet preferences (16). These findings underscores that understanding and working with patient preferences is nuanced but can play a vital role in optimizing treatment engagement and effectiveness.

Evidence from psychiatric outpatient samples further highlights the need to consider patient preferences in clinical decision-making. A large cross-sectional study with 677 consecutive psychiatric outpatients found that while most preferred a collaborative role, the majority experiences a passive role during treatment decisions (17). This mismatch between desired and experienced participation underscores the importance of actively engaging patients in shared decision making processes to better align treatment delivery with individual preferences. A national study in England found that most patients expressed preferences regarding psychological treatment (18). Crucially, patients whose preferences were not accommodated reported significantly poorer outcomes compared to those whose preferences were met.

Several meta-analyses have investigated the impact of accommodating patient preferences in mental health care, yet their conclusions vary depending on the study design, targeted outcomes, and populations examined. Overall, preference accommodation tends to be associated with reduced dropout rates and, to a lesser extent, with improved clinical outcomes. For example, Swift, Callahan (19) conducted the most comprehensive meta-analysis to date, including over 16,000 patients, and found that receiving a preferred treatment significantly reduced dropout (OR = 1.79) and improved treatment outcomes (*d* = 0.28). Johnson, Radunz (20) confirmed beneficial effects for depression (*d* = 0.17), as well as significantly reduced dropout across depression, anxiety, and eating disorders (OR = 1.46). However, not all reviews found consistent effects. Windle, Tee (21) reported a clear association between preference accommodation and reduced dropout and improved therapeutic alliance (*d* = 0.48), but no significant effect on symptom improvement or global functioning. Similarly, Eigenhuis, van Buuren (22) found significant effects of preference matching on satisfaction and adherence, but no consistent impact on clinical symptom outcomes. Taken together, these findings suggest that while preference accommodation may not consistently enhance symptom-based outcomes, it reliably improves engagement, adherence, and the therapeutic alliance—all of which are critical mechanisms for successful treatment.

The question remains what treatment people prefer when they have a mental disorder. Despite the availability of evidence-based treatment options, there is growing interest in understanding which treatments are preferred by the public. Several studies have demonstrated a clear preference for psychotherapy over pharmacological treatment. A meta-analysis by McHugh, Whitton (23) found that approximately 75% of respondents preferred psychological treatment – a finding consistent across both treatment-seeking and non-treatment-seeking samples. The included studies primarily focused on depression and anxiety disorders, with a smaller number examining posttraumatic stress disorder, obsessive compulsive disorder or general mental health conditions. Representative surveys from Germany similarly showed that psychotherapy is generally viewed as the preferred option (24, 25). Even among male psychiatric outpatients, a group often assumed to be less open to psychotherapy, a strong preference for psychological interventions was observed (26). However, much of the available evidence is outdated and may not fully reflect recent developments. In recent years, public discourse around mental health has changed substantially. Increased media coverage, digital health campaigns, and broader destigmatization efforts have contributed to a growing social awareness and openness regarding psychological distress. This is particularly true in the wake of the COVID-19 pandemic, which brought mental health challenges to the forefront of public consciousness. As a result, treatment preferences may have shifted over time – influenced not only by greater knowledge and acceptance, but also by firsthand experience of mental strain and increased demand for support services. Yet, to date, most empirical work on treatment preferences relies on data collected before these developments, potentially overlooking current trends and expectations. Against this backdrop, the present study offers a timely update, aiming to capture contemporary treatment preferences within the general population and explore how they align with current guideline recommendations.

To our knowledge, this is the first study to directly compare disorder-specific treatment preferences with guideline-based first-line recommendations in the general population. By focusing on contemporary data collected after major shifts in public mental health discourse, this study provides an up-to-date perspective on whether current preferences support or challenge guideline recommendations. We hypothesize that (a) psychotherapy will be the most preferred treatment modality across disorders; (b) preference strength will vary by diagnosis, with the highest preference for psychotherapy in depression and anxiety and more balanced preferences in psychotic disorders; and (c) persons with previous treatment experience will show preferences more aligned with guidelines recommendations.

## Methods

### Sample characteristics

A total of 2,134 persons initially participated in the online survey. After excluding incomplete or invalid cases (*n* = 9), the final sample comprised *N* = 2,125 adults. The sample can be considered broadly representative of the general population in Germany in terms of age and gender distribution (51.6% men, 48.2% women, 0.3% diverse). Recruitment took place between May and August 2024 via the online panel provider and market research company Bilendi. The participation was voluntary and anonymous with a monetary compensation in accordance with the panel provider´s standard incentive scheme (cash or points, approximately 0.50–0.80€, depending on survey length). Inclusion criteria was a minimum age of 18 years. The age ranged between 18 and 69 years, with the majority falling within the 30 to 69-year age range (**Table 1**). Sociodemographic characteristics of the final sample are summarized in **Table 1**.

**Table 1:** Sociodemographic characteristics of the final sample (N = 2,125).

Characteristics	Category	*n* (%)
Age	18–29 years	294 (13.8%)
	30–49 years	893 (42.0%)
	50–69 years	938 (44.1%)
Marital status	With partner	1,459 (68.7%)
	Without partner	666 (31.3%)
Income	< 2,000 €	548 (25.8%)
	2,000–3,999 €	1,014 (47.7%)
	≥ 4,000 €	563 (26.5%)
Job	Employed (all types)	1,376 (64.8%)
	Self-employed/freelancer	154 (7.2%)
	Student/apprentice	113 (5.3%)
	Retired	289 (13.6%)
	Other (homemaker, unemployed, etc.)	193 (9.1%)

### Measures

The survey was collaboratively developed by research teams from the University Hospital of Munich (LMU) and the University Medical Centre Hamburg-Eppendorf (UKE) and administered online via Qualtrics. It included questions on sociodemographic characteristics, previous mental health treatment, and preferences for various treatment options. Participants were also asked about personal experience with outpatient or inpatient mental health care, employment in the healthcare sector, and whether they provide treatment or advice to persons with mental health conditions in their professional role.

The survey included a vignette-like battery assessing agreement with 11 different treatment options across eight mental disorders. The hypothetical mental health conditions that were presented were depression, anxiety disorder, obsessive-compulsive disorder, eating disorder, post-traumatic stress disorder, somatoform disorder, personality disorder, and psychosis. The treatment options were selected to reflect a broad range of commonly considered treatment and coping strategies, including both formal treatment options and informal approaches. Digital interventions were not included as a single category due to their heterogeneity, and a more detailed differentiation was beyond the scope of the present study. Therefore, the following treatment options were presented: psychopharmacology, outpatient psychotherapy, combined treatment with psychotherapy and psychopharmacology, alternative treatments (e.g., naturopathy, herbal or homeopathic remedies), talking to friends or family, physical exercise, self-help literature, internet-based self-help programs, local support group, inpatient treatment in a psychiatric hospital and inpatient treatment in a psychosomatic clinic. While most psychiatric hospitals primarily focus on acute crisis management and severe mental illnesses — often using a combination of pharmacological treatment and crisis intervention — psychosomatic clinics typically provide psychotherapeutically oriented care, including frequently individual and group therapy, for mood, anxiety, somatoform, trauma-related, eating and personality disorders. However, this distinction was not explicitly explained to participants within the survey. Agreement with each option was rated on a 5-point Likert-Scale ranging from 1 = “I would not consider this option” to 5 = “I would consider this option”. For anxiety and obsessive-compulsive disorder, participants who indicated willingness to undergo psychotherapy were further asked to choose between a conventional weekly format (11 weeks) or a compact format (3.5 consecutive days), reflecting different delivery formats of exposure-based treatments.

### Statistical analyses

All analyses were conducted using R version 4.4.3 (27). First, linear mixed-effects models were used to examine whether participants’ agreement with different treatment options (psychotherapy, psychopharmacology, combination) varied depending on the mental disorder (e.g., depression, anxiety, OCD, etc.). The model included fixed effects for disorder, option, and their interaction (disorder × option), and a random intercept for each participant to account for repeated measurements. The dependent variable was agreement with the treatment option on a 5-point Likert scale.

Second, based on the estimated marginal means from the linear mixed-effects model, custom contrasts were calculated using the emmeans package to compare preference for psychotherapy versus psychopharmacology within each disorder (28). This allowed for a direct comparison of the relative acceptability of psychotherapy and psychopharmacology across diagnostic categories. For all models, significance was determined at *p* <.05. Effect sizes were calculated using partial eta squared (29). Only complete cases were included in the analyses. As no missing data were present in the variables included in the models, this did not lead to any exclusions, and the sample size remained constant across models (*N* = 2,125).

In a third step, we conducted a multinomial logistic regression to examine predictors of participants’ general treatment preference (i.e., psychotherapy, psychopharmacology or a combination of both). The dependent variable was based on a specific survey item assessing self-reported preference for treatment in the case of a hypothetical mental disorder in general. The reference category was set to psychotherapy. Predictors included age group, gender, previous treatment experience (yes/no), and employment in the healthcare sector (yes/no). The model was estimated using the multinom() function from the nnet package (30). For each comparison (e.g., combination vs. psychotherapy, psychopharmacology vs. psychotherapy), log-odds coefficients, standard errors, *z*-values, and two-tailed *p*-values were computed. Significance was determined at *p* <.05.

## Results

Of the 2,125 participants, 35.5% (*n* = 754) reported that they had previously received treatment for a mental disorder. Regarding the treatment setting, 28.0% (*n* = 595) had received outpatient treatment, while 12.4% (*n* = 264) had been treated in a psychosomatic inpatient clinic, and 10.6% (*n* = 225) in a psychiatric inpatient clinic (multiple responses possible). Additionally, 12.6% (*n* = 269) of participants were employed in the healthcare sector. Among them, 6.8% (*n* = 145) reported that they personally treated persons with mental health problems, and 21.7% (*n* = 461) reported that they advise such persons at least occasionally. In terms of general treatment preferences, 40.5% of participants preferred psychotherapy, 40.6% preferred a combination of psychotherapy and psychopharmacology, and 5.7% preferred psychopharmacology alone. An additional 13.2% indicated a preference for alternative treatment methods (e.g., naturopathy, herbal remedies). Multiple responses were possible. When asked about their preference regarding inpatient treatment settings, 11.2% preferred a psychiatric hospital, 23.1% preferred a psychosomatic clinic, 35.8% expressed no preference between the two, and 30.0% stated they would not consider inpatient treatment under any circumstances.

**Figure 1:**
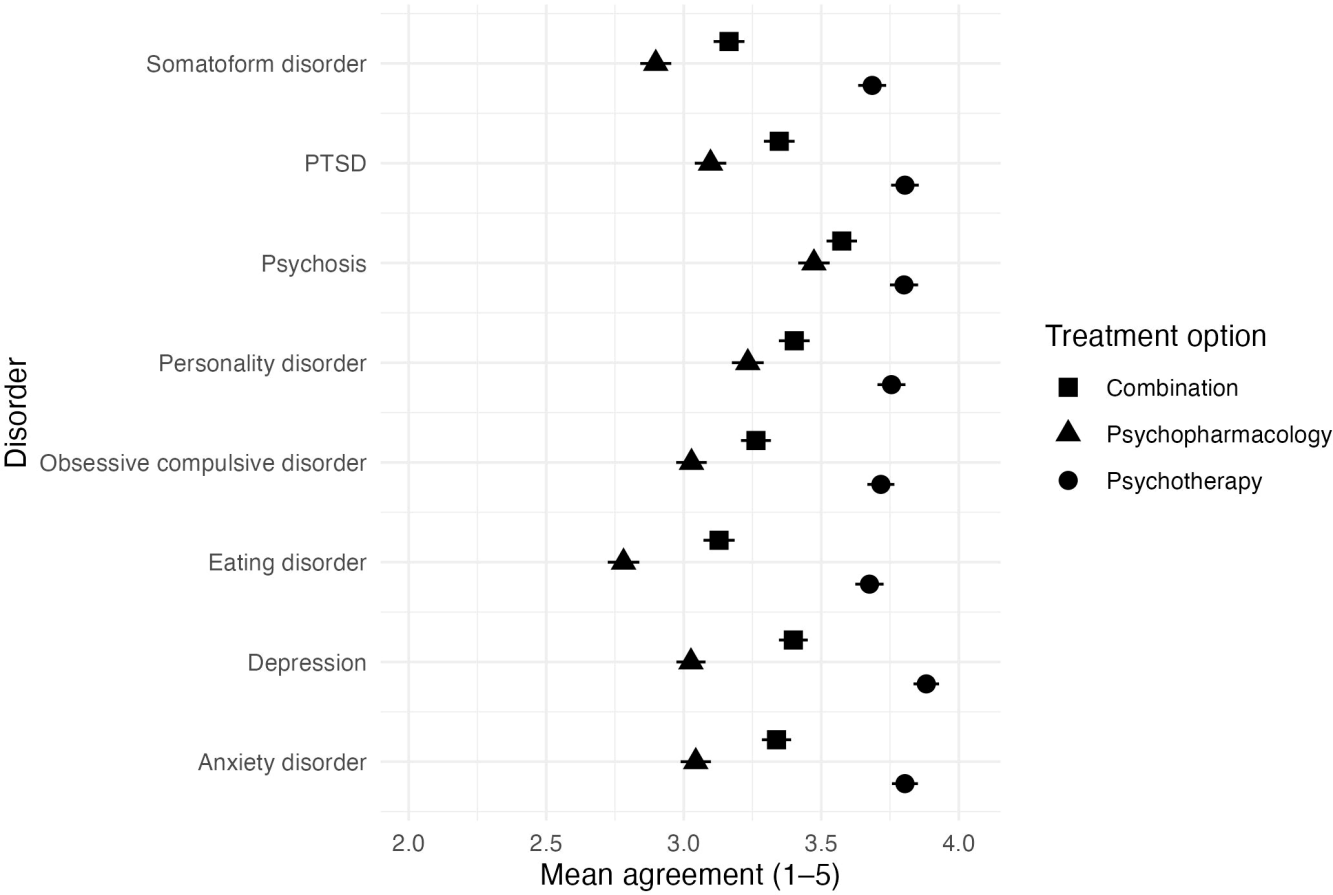
Mean agreement with guideline-based treatment options across mental disorders. Mean agreement ratings (1–5 scale) for psychotherapy, psychopharmacology, and their combination are shown for different mental disorders. Circles represent psychotherapy, triangles represent psychopharmacology, and squares represent combination treatment. Error bars represent 95% confidence intervals.

First, a linear mixed-effects model was conducted to examine whether agreement with evidence-based treatment options (psychotherapy, psychopharmacology, combination therapy) differed across mental health disorders. **Figure 1** displays the estimated marginal means by treatment option and disorder. The results revealed significant main effects of treatment option (*p* <.001), disorder (*p* <.001), and a significant interaction between treatment option and disorder (*p* <.001). Overall, psychotherapy received the highest average agreement ratings, particularly for depression (*M* = 3.88, *SE* = 0.03, 95% CI [3.82, 3.94]). In comparison, combination therapy (*M* = 3.40, *SE* = 0.03, 95% CI [3.34, 3.46]) and especially psychopharmacology (*M* = 3.03, *SE* = 0.03, 95% CI [2.97, 3.09]) were rated significantly lower (*p* <.001). Importantly, the extent of agreement varied by disorder. For example, agreement with psychopharmacology was considerably higher for psychosis (*b* = 0.53, *p* <.001) and personality disorders (*b* = 0.33, *p* <.001) compared to depression. Conversely, agreement with psychotherapy was slightly lower for most disorders compared to depression but remained relatively high overall. Treatment option had a large effect (*partial η²* = .10), while disorder (*partial η²* = 0.02) and the interaction (*partial η²* = 0.01) showed only small effects.

**Figure 2:**
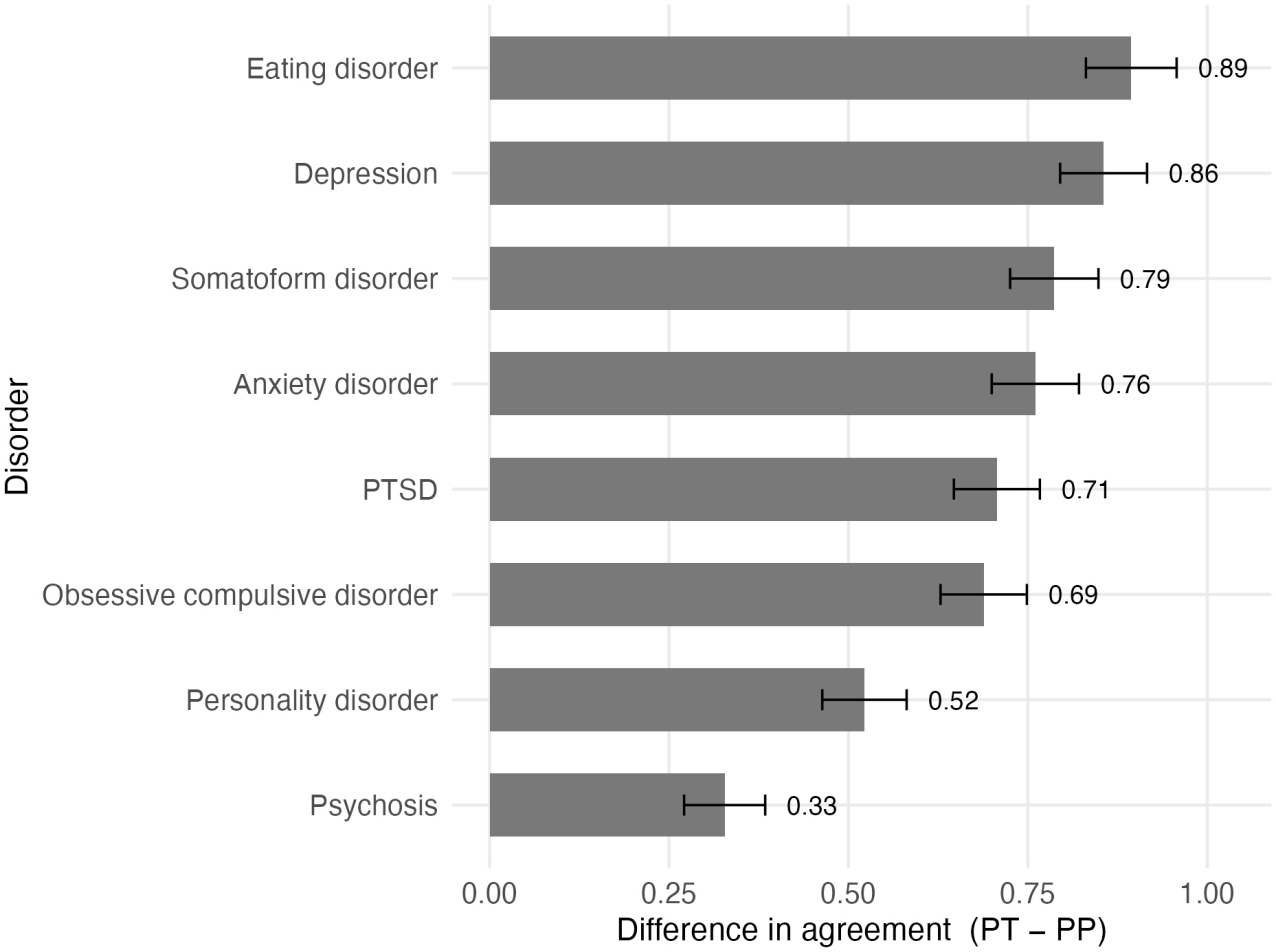
Differences in preference for psychotherapy (PT) versus psychopharmacology (PP) across mental disorders. Differences in mean agreement ratings (psychotherapy minus psychopharmacology, 1–5 scale) are shown for each disorder. Positive values indicate a stronger preference for psychotherapy. Error bars represent 95% confidence intervals.

To further explore the differences in agreement with psychotherapy or psychopharmacology as therapy, pairwise contrasts were computed between psychotherapy and psychopharmacology within each disorder, based on the estimated marginal means from the mixed model. Across all disorders, agreement with psychotherapy was significantly higher than with psychopharmacology (*p* <.001). The difference varied by disorder (**Figure 2**). The largest differences in favour of psychotherapy were observed for eating disorders (*ΔM* = 0.55, *SE* = 0.03, *p* <.001), somatoform disorders (*ΔM* = 0.52, *SE* = 0.03, *p* <.001), and anxiety disorders (*ΔM* = 0.47, *SE* = 0.03, *p* <.001). The smallest difference was found for psychosis (*ΔM* = 0.23, *SE* = 0.03, *p* <.001), although psychotherapy was still rated more favourably than psychopharmacology.

While the previous analyses focused on disorder-specific differences in treatment agreement, we additionally explored which individual characteristics predicted a general preference for psychotherapy, psychopharmacology, or combined treatment using multinomial logistic regression, based on a specific survey item assessing self-reported treatment preference in the case of having any mental disorder. Results showed that female participants were significantly less likely to prefer pharmacological treatment over psychotherapy (*b* = –0.63, *p* = .002), as were participants with prior treatment experience (*b* = –0.39, *p* = .048) and those working in the healthcare sector (*b* = –0.61, *p* = .016). In contrast, preference for combination treatment was not significantly associated with any predictor variables (all *p* >.05). Notably, participants with previous treatment experience were significantly more likely to prefer alternative treatment methods over psychotherapy (*b* = 1.18, *p* <.001).

Finally, we explored agreement with additional treatment options such as alternative treatments, exercise, family support, self-help materials, and online programs. Ratings varied substantially by disorder and treatment type. Exercise and family support received high agreement, while books and online resources were rated more moderately (**Supplementary Figure 1**).

## Discussion

### Summary of key findings

The present study reveals a strong overall preference for psychotherapy across a range of mental disorders. Psychotherapy received the highest average agreement, especially for depression, but was also associated with the highest reported preference across all disorders. Although psychopharmacology was comparatively more accepted for psychotic disorders and personality disorders, it was still rated lower than psychotherapy. We observed a large effect of treatment type, indicating a substantial difference in preference between psychotherapy and psychopharmacology. In contrast, the effect of disorder type, as well as the interaction between disorder and treatment type was small. The most pronounced differences in favour of psychotherapy were found for eating disorders, somatoform disorders, and anxiety disorders.

Women were less likely to prefer psychopharmacology than men, however, both genders overall reported a preference for psychotherapy. Furthermore, persons with prior mental health care experience were more likely to indicate a preference for alternative treatment options beyond psychotherapy and psychopharmacology.

### Comparison to prior research

Approximately one-third of the participants reported having received treatment for mental health problems in the past. This rate is notably higher than the 24.5% reported by Mack, Jacobi (31) in a representative German sample of 4,483 participants. The discrepancy may indicate an increasing trend in mental health service utilization in recent years, but it may also reflect a true rise in the prevalence of mental disorders. This interpretation is supported by further evidence: Several studies have documented relatively low but steadily increasing mental health service utilization rates among persons with mental health problems in Germany (31, 32). In our sample, 10.6% reported having been treated in a psychiatric hospital, and 12.4% in a psychosomatic clinic. In comparison, Mack, Jacobi (31) reported that only 4.8% had received treatment in a psychiatric hospital/department and 5.9% in a psychotherapeutic or psychosomatic clinic. This notable increase may reflect a growing demand for inpatient mental health care, possibly due to limited availability of outpatient psychotherapy and long waiting times (33). In recent years inpatient treatment capacities in psychosomatic clinics have also expanded considerably.

The strong preference for psychotherapy observed in our study is in line with prior national and international research. For instance, McHugh, Whitton (23) reported in their meta-analytic review that adults were nearly three times more likely to prefer psychological over pharmacological treatment for depression and anxiety. This preference was consistent across subgroups, though somewhat more pronounced in younger and non-treatment-seeking persons. Our results mirror this pattern except the subgroup results: psychotherapy was the most favoured treatment across disorders, especially for depression and anxiety, whereas psychopharmacology was viewed more favourably only in the context of psychotic and personality disorders. Similarly, a representative German study by Riedel-Heller, Matschinger (24), using a forced-choice ranking design, found that the majority of respondents chose psychotherapy as the preferred first-line treatment for both depression and schizophrenia. Psychopharmacological treatment was far less frequently recommended, even when respondents recognized the condition as a mental illness—highlighting a general scepticism toward psychopharmacology as a primary treatment option. More recent data from Van der Auwera, Schomerus (25) confirm that psychotherapy enjoys high approval across all age groups, while attitudes toward pharmacological treatment remain more critical, particularly among younger persons. Our study replicates and extends this general tendency and adds nuance by showing that women and individuals with previous mental health care experience were less likely to prefer pharmacological treatment over psychotherapy, while individuals with previous treatment experience were more likely to indicate a preference for alternative treatment methods over psychotherapy. This finding is in line with previous research showing that complementary and alternative treatment approaches are commonly used among persons with depression (34).

### Alignment of public preference and clinical guidelines: implications for mental health care

The strong preference for psychotherapy observed in our study is largely consistent with current international and national clinical guidelines, which recommend psychotherapy as a first-line treatment for a broad range of mental disorders. For instance, psychotherapy is prioritized for depression (mild to moderate), anxiety, post-traumatic stress disorder, eating disorders, somatoform disorders, and personality disorders, with psychopharmacology typically reserved for severe or comorbid cases (5–13). Our findings on reported preferences mirror these recommendations. However, these findings reflect hypothetical preferences and should be interpreted with caution when drawing conclusions about real-world treatment decisions. Psychopharmacology was more often preferred only in the context of psychotic disorders, which aligns with guidelines recommending a combined approach (14, 15).

Given this high degree of alignment between public preferences and guideline recommendations, integrating both perspectives into clinical decision-making appears both feasible and potentially essential. As highlighted by Swift, Mullins (35), accommodating patient preferences enhances treatment initiation and outcomes and reduces dropout rates. In parallel, adherence to evidence-based guidelines is associated with faster symptom reduction and improved overall outcomes (4). Thus, combining guideline-based recommendations with patients’ preferences can enhance both the effectiveness and acceptability of treatment.

However, real-world service provision does not yet fully reflect either preferences or guidelines. As (32) demonstrated, only about one third of persons with current depressive symptoms in Germany receive psychotherapeutic or psychiatric care. Regional disparities in provider density further exacerbate this gap (32). While reforms such as the updated psychotherapy directive and stepped-care approaches aim to lower access barriers, significant challenges remain (36). This underscores the need for health care systems to expand access to psychotherapeutic services to better meet patient expectations and improve treatment outcomes.

### Strengths and limitations

One of the key strengths of this study lies in its large and diverse sample from the general population. In contrast to many prior studies that have solely examined public treatment preferences, our approach also integrated national clinical guidelines, thereby enhancing the clinical relevance of the findings. Furthermore, the differentiation across multiple mental disorders allows for more disorder-specific insights and increases the practical applicability of the results, for example in the context of shared decision making or psychoeducation about pharmacological and psychotherapeutic treatment options. Another key strength of the current study is its timeliness. By collecting data in a post-pandemic context, it reflects recent societal shifts in how mental health is perceived and discussed. The COVID-19 pandemic significantly increased public engagement with topics related to psychological well-being, which may have influenced both awareness of treatment options and personal attitudes toward seeking help. As such, the present findings provide an important update to earlier studies conducted in a different societal climate. They offer a contemporary snapshot of mental health treatment preferences that is highly relevant for current service planning and health communication strategies.

However, several limitations should be acknowledged. Although Bilendi follows strict procedures to ensure data quality and reduce bias including diverse recruitment channels, some biases might persist due to online access requirements, self-selection, and potential survey fatigue. Moreover, the proportion of participants with previous mental health treatment was higher than in representative surveys, which may indicate a growing use of mental health services but could also reflect a self-selection bias, with persons who have treatment experience being more likely to participate or to respond to mental health-related questions. Additionally, the understanding of the diagnostic labels and treatment options used in the survey may have varied across respondents, which could lead to some conceptual ambiguity regarding the conditions being rated. Furthermore, the range of treatment options presented was necessarily limited and did not include certain emerging approaches, such as digital health applications, which may have influenced reported preferences. Children, adolescents, and older adults were not included, which limits the generalizability of the findings across the entire lifespan. Finally, the self-constructed questionnaire in the vignette-based study design and the simplified descriptions of mental disorders may not fully reflect the complexity of real-world diagnoses and treatments, as responses capture hypothetical preferences that may not translate directly into actual treatment decisions. Moreover, as the study was conducted in Germany, the findings may not be generalizable to other countries or healthcare systems. In particular, the German healthcare system is characterized by broad insurance coverage and a sectorally structured mental healthcare system, including inpatient, outpatient, day care, rehabilitation, and complementary regional services. These features may have influenced the observed preferences, for example by increasing the acceptability of psychotherapy, which is typically covered by health insurance, relative to other treatment options (36).

## Conclusion

In conclusion, the findings highlight the potential importance of aligning mental health care services with both public preferences and clinical guideline recommendations. Psychotherapy is widely accepted and preferred across most disorders, reflecting both evidence-based recommendations and public expectations. Psychotherapy may need to be more promptly and widely accessible as psychopharmacology – at least for those disorders where it is recommended as a first-line treatment. To improve treatment outcomes, future efforts should focus on reducing structural barriers to accessing psychotherapy and on promoting informed, preference-sensitive decision making. Further research should explore how preferences translate into actual treatment utilization and how they evolve across different populations and life stages. Considering the high preference for psychotherapy and its recommendation as a first-line, evidence-based treatment in clinical guidelines for many mental disorders, it is also important to investigate how these preferences can be met in practice – particularly with regard to ensuring appropriate access, continuity, and eventual discharge from the system.

## Data Availability

The raw data supporting the conclusions of this article will be made available by the authors, without undue reservation.
