# Supplementary material for: What if I needed help? Population preferences for first-line mental health treatments in the post-COVID time
Source: Front Psychiatry. 2026 May 28;17:1843041. doi: 10.3389/fpsyt.2026.1843041 (PMC13254198; doi:10.3389/fpsyt.2026.1843041)
Supplement: Supplementary file 1 [file Supplementaryfile1.docx]

Supplementary Material

**Supplementary Figure 1.**


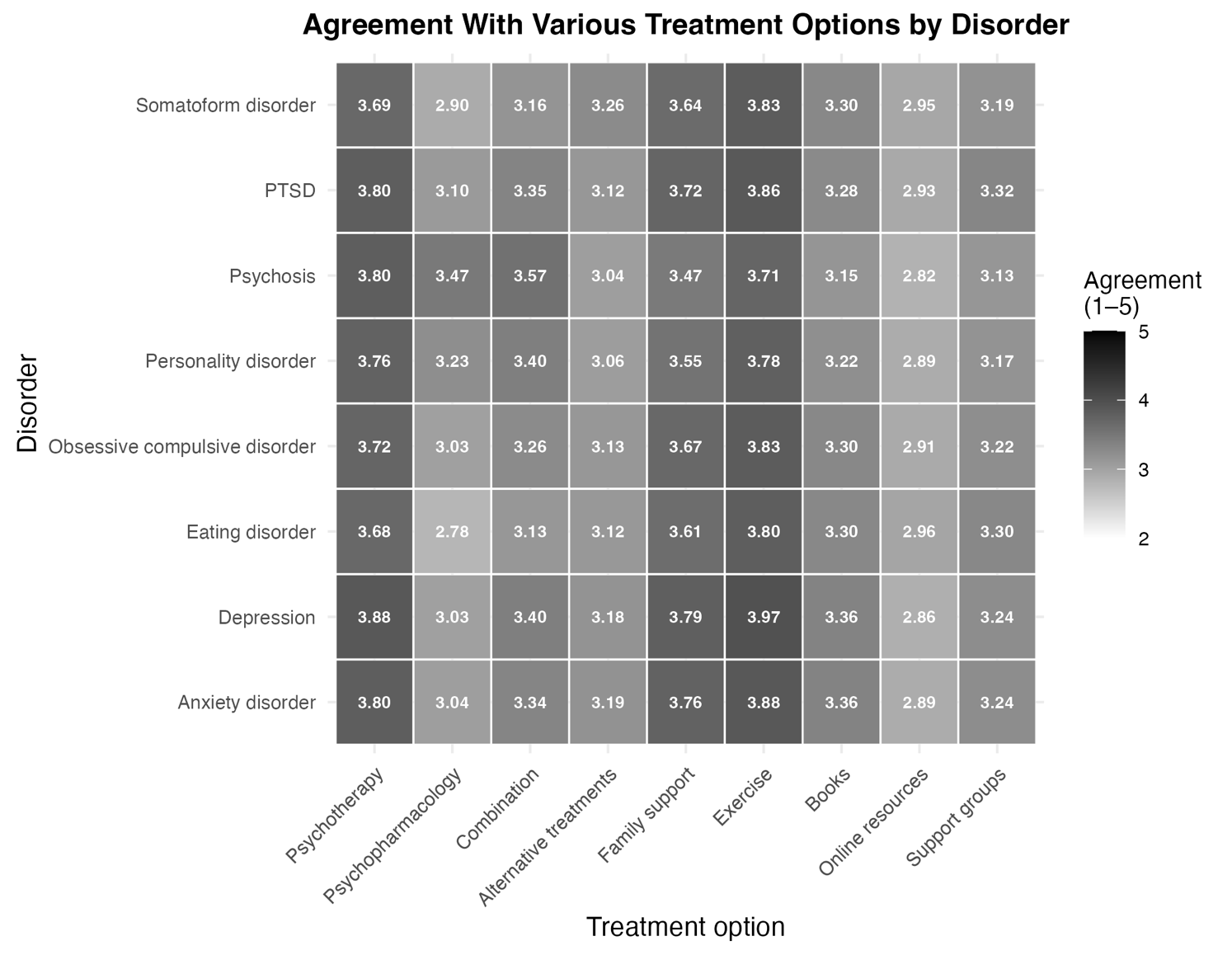


**Supplementary Figure 1.** Mean agreement ratings (scale 1–5) for different treatment options across somatoform disorders, post-traumatic stress disorder, psychosis, personality disorder, obsessive-compulsive disorder, eating disorder, depression, and anxiety disorder. Higher values indicate greater agreement with the respective treatment option. Darker shading represents higher agreement, whereas lighter shading indicates lower agreement.
